# Editorial: Advanced analytical techniques for heavy metal(loid)s speciation in soil, crop, and human samples

**DOI:** 10.3389/fchem.2023.1151371

**Published:** 2023-02-03

**Authors:** Ping Xiang, Yong-He Han, Hong-Bo Li, Peng Gao

**Affiliations:** ^1^ Institute of Environmental Remediation and Human Health, School of Ecology and Environment, Southwest Forestry University, Kunming, China; ^2^ College of Environmental and Resource Sciences, Fujian Normal University, Fuzhou, China; ^3^ School of Environment, Nanjing University, Nanjing, China; ^4^ Department of Environmental and Occupational Health, University of Pittsburgh, Pittsburgh, PA, United States

**Keywords:** heavy metal(loid)s, speciation analysis, bioaccessibility and bioavailability, immobilization, sensor

Heavy metal pollution is a worldwide environmental problem that poses a threat to human health. The growing population and demand for food result in excessive metal(loid)s releases into the environment, contaminating soil and water and further affecting food crops. The health effects of excessive metal(loid)s vary based on the type of metal(loid)s and its speciation. Thus, studying metal(loid) speciation in soil, food, and human specimens is crucial to understanding the health risks and determining factors like bioavailability, toxicity, and biological responses. This field of research involves interdisciplinary analysis approaches including experimental and computational techniques.

Trace metal(loid)s in the environment, such as soil, water, food, and biological samples, are difficult to detect accurately due to their varying concentrations and properties. Understanding their speciation is crucial for evaluating their toxic risk and how they excrete from the human body if overexposure. Knowing the composition and speciation of metal(loid)s can help researchers better assess their health risks. The current collection of research emphasizes some interesting and novel findings using advanced analytical techniques for metal(loid)s. The editors hope it will spark further interest and new studies in this field.

Overall, various advanced analytical techniques can be used for the speciation of heavy metal (loid)s in environmental and biological samples. These include inductively coupled plasma coupled with optical emission spectrometry or mass spectrometry, X-ray absorption spectroscopy, and molecular spectroscopy. Furthermore, several kinds of chromatography are used for separating the heavy metal (loid)s from the samples before detection, different microscopy are used to study the morphology and distribution of heavy metal (loid)s in samples, and biotechnology techniques are also useful in the detection, immobilization, and removal of heavy metal (loid)s from environmental and biological samples. However, it is essential to note that different techniques may be more or less appropriate depending on the specific sample and research question. Selecting the correct technique or combination of techniques to obtain the most accurate and relevant results is critical.

This Research Topic contains the Research Topic of four original contributions dedicated to the different aspects related to the advanced analytical techniques for heavy metal (loid)s speciation as well as biomolecules in soil, crop, and human samples. Hand-to-mouth activity is considered to be the main way for children to come into contact with contaminated soil, and bioavailability is an important factor affecting their health risk. To reduce soil arsenic (As) risk to humans by oral exposure, Chen et al. treated two contaminated soil samples with nanoscale zero-valent iron (nZVI) for 56 days and investigated its effects on arsenic bioaccessibility and bioavailability *in vitro* and *in vivo*, respectively, and they found nZVI impacted As bioaccessibility and relative bioavailability (RBA). Since As-RBA can assess the effects of nZVI on human arsenic exposure more accurately, the author pointed out that it is necessary to develop a suitable *in vitro* assay to predict As-RBA in nZVI-amended soils. Besides soils, developing novel technologies to remove metal irons from contaminated water and decrease their adverse effect are also urgent to be solved. Chen et al. developed a gold nanoparticles-deoxyribozymes-labeled filter paper as a portable sensor to detect the total concentrations of heavy rare Earth (gadolinium, terbium, and dysprosium) ions with similar chemical properties. The authors claimed this technology provides a new sensor option to real-time monitor the presence of heavy rare Earth elements in wastewater from industrial facilities. Besides detection, a study related to the removal of heavy metal (loid)s is included in this Research Topic as well. Lan et al. employed 3D-printed cylindrical capsules as a Chlorella pyrenoidosa immobilization device to remove lead ions contamination from water. As a result, the developed capsules showed a high adsorption capacity for removing lead and present a promising recovery property. In addition, accurately determining molecules in biological samples could be more challenging than that of soils or waters due to various Research Topic such as complex matrix effects. As such, Zhang et al. developed a straightforward and sensitive ultra-high-performance liquid chromatography-tandem mass spectrometry method to quantify 17 endogenous adrenal corticosteroid hormones in human plasma samples. The authors stated that the application of this method has great potential in the simultaneous measurement of steroid hormones and could improve the diagnostic efficiency of congenital adrenal hyperplasia.

In summary, the editors hope that this Research Topic will be of great interest to the *Frontiers in Chemistry* readers and will inspire significant progress in the field of advanced analytical techniques for heavy metal (loid)s.

